# Reducing inter-observer variability and interaction time of MR liver volumetry by combining automatic CNN-based liver segmentation and manual corrections

**DOI:** 10.1371/journal.pone.0217228

**Published:** 2019-05-20

**Authors:** Grzegorz Chlebus, Hans Meine, Smita Thoduka, Nasreddin Abolmaali, Bram van Ginneken, Horst Karl Hahn, Andrea Schenk

**Affiliations:** 1 Fraunhofer Institute for Digital Medicine MEVIS, Bremen, Germany; 2 Diagnostic Image Analysis Group, Department of Radiology and Nuclear Medicine, Radboud University Medical Center, Nijmegen, The Netherlands; 3 University of Bremen, Medical Image Computing Group, Bremen, Germany; 4 Department of Radiology, Städtisches Klinikum Dresden, Dresden, Germany; 5 Jacobs University, Bremen, Germany; University of Alberta, CANADA

## Abstract

**Purpose:**

To compare manual corrections of liver masks produced by a fully automatic segmentation method based on convolutional neural networks (CNN) with manual routine segmentations in MR images in terms of inter-observer variability and interaction time.

**Methods:**

For testing, patient’s precise reference segmentations that fulfill the quality requirements for liver surgery were manually created. One radiologist and two radiology residents were asked to provide manual routine segmentations. We used our automatic segmentation method Liver-Net to produce liver masks for the test cases and asked a radiologist assistant and one further resident to correct the automatic results. All observers were asked to measure their interaction time. Both manual routine and corrected segmentations were compared with the reference annotations.

**Results:**

The manual routine segmentations achieved a mean Dice index of 0.95 and a mean relative error (RVE) of 4.7%. The quality of liver masks produced by the Liver-Net was on average 0.95 Dice and 4.5% RVE. Liver masks resulting from manual corrections of automatically generated segmentations compared to routine results led to a significantly lower inter-observer variability (mean per case absolute RVE difference across observers 0.69%) when compared to manual routine ones (2.75%). The mean interaction time was 2 min for manual corrections and 10 min for manual routine segmentations.

**Conclusions:**

The quality of automatic liver segmentations is on par with those from manual routines. Using automatic liver masks in the clinical workflow could lead to a reduction of segmentation time and a more consistent liver volume estimation across different observers.

## Introduction

Total liver volume plays an essential role in planning liver interventions such as liver surgery or radioembolization and in therapy response assessment [1, 2]. Manual liver segmentation is a tedious and time-consuming task, which also is prone to inter-observer variability, in particular for MRI data. Automation of the liver contouring process allows for shorter segmentation times and reduction of measurement subjectivity [3].

Recently, several approaches for automatic liver segmentation in MRI have been proposed. A 3D liver model guided by a precomputed probability map was used by Bereciartua et al. [4], which achieved a mean Dice of 0.90 for patients with healthy livers. Le et al. used a histogram-based liver segmentation with a subsequent geodesic active contour refinement step and reported a mean Dice of 0.91 [5]. Huyhn et al. proposed a method based on watershed transformation and active contours, which achieved a mean Dice of 0.91 [6]. Christ et al. trained a fully convolutional neural network for this problem, which produced liver masks with a mean Dice of 0.87 [7].

A related problem of automatic liver segmentation in CT images has received more attention recently compared to segmentation in MRI data thanks to the Liver Tumor Segmentation Challenge (LiTS) organized in 2017 [8]. Top-ranking submissions used deep learning based segmentation approaches including hierarchical models for coarse-to-fine segmentation [9], 3D networks combining original image data with features derived from 2D models [10] and orthogonal 2D networks [11]. Other well-established algorithms for CT liver segmentation employ statistical shape models to describe plausible shape variations [12, 13].

Whereas automatic methods produce liver segmentations of a quality close to that of experts, they can still fail in some cases, thus requiring manual corrections. As reported in [6], major segmentation errors are caused by leakage into neighboring organs (heart, colon, stomach, or kidneys) or by lesions located near the organ’s boundary. None of the recently published MRI segmentation methods was evaluated with respect to the required time to correct such major errors, which should be considered when assessing the overall utility of an automatic method.

The contribution of this paper is twofold. First, we provide a validation of our automatic method in MRI based on convolutional neural networks dubbed Liver-Net by comparing its results with manual routine segmentations performed by clinicians. Second, we report on segmentation errors of Liver-Net and required correction time by clinical users. Additionally, we investigate the influence of automatic liver proposals on the inter-observer variability with respect to the estimated total liver volume.

## Materials and methods

### Data

For our study, we used DCE-MRI data of 83 patients with primary liver cancer and/or liver metastases that were scheduled for selective internal radiation therapy (SIRT). Image data was acquired at Städtisches Klinikum Dresden, Germany, on a 3T MRI scanner (Discovery, GE Healthcare Systems, USA). The image in-plane resolution ranged from 0.74 to 1.76 mm and the slice thickness from 2 to 5 mm. Segmentations were performed on the late hepatocellular phase acquired 15 min after intravenous injection of the contrast agent (Gd-EOB-DTPA, Bayer, Germany). We randomly selected 62 cases for training and optimization of our automatic method. The remaining 21 cases were left out for evaluation.

For all patients, reference segmentations were performed by two radiologist assistants with over ten years of experience in a precise segmentation according to liver-surgery planning requirements. The contours were drawn using a semi-automatic tool [14] and reviewed by a radiologist.

Imaging data for this study was evaluated at Städtisches Klinikum Dresden after approval by the ethics committee of Sächsiche Landesärztekammer (EK-BR-79/16-1 / 118834). All patients gave written informed consent. The study did not include minors.

### Automatic segmentation method

#### Architecture

**Fig 1:**
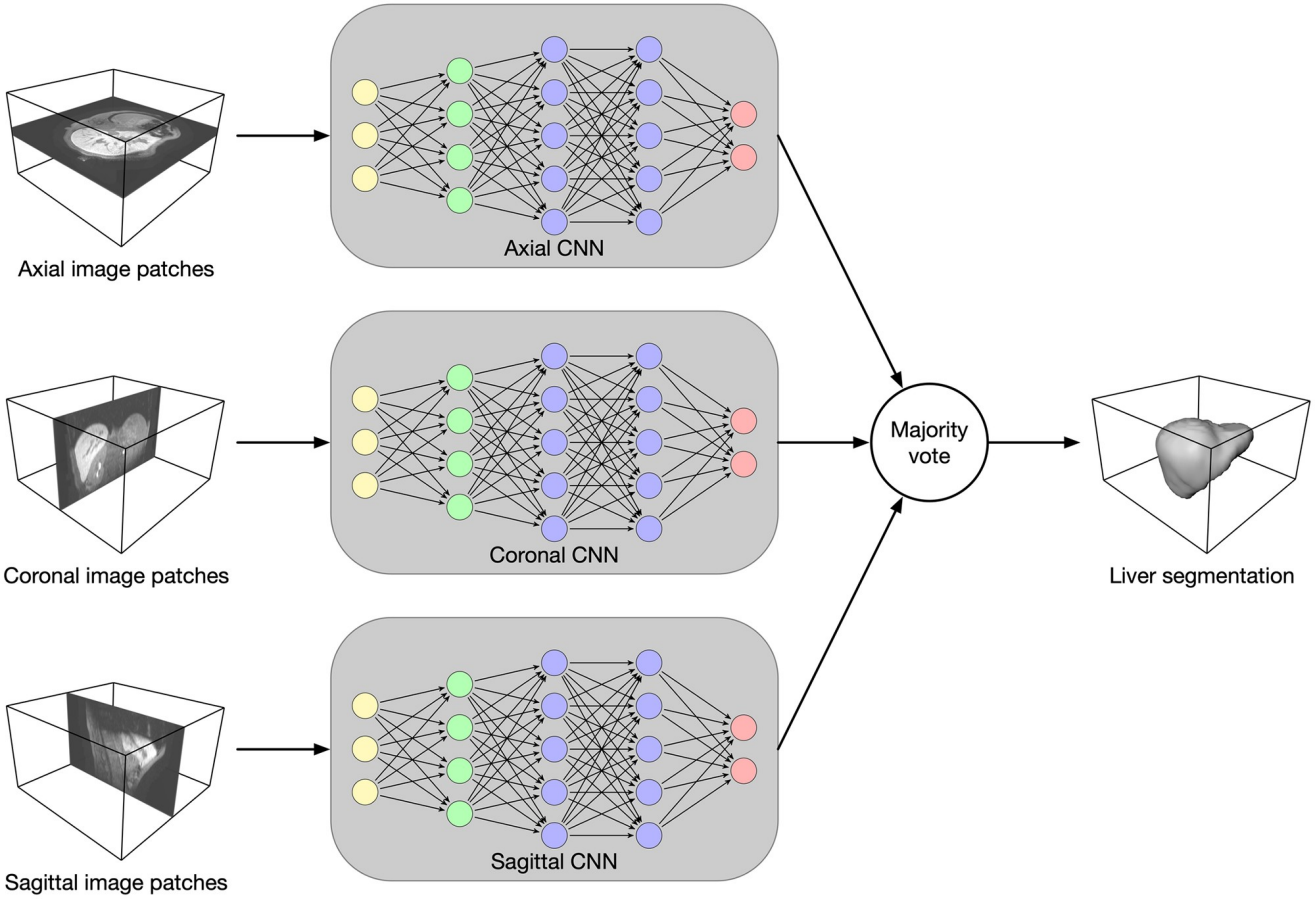
Overview of the Liver-Net method. Three 2D U-net-like models analyze axial, coronal and sagittal image patches. The final segmentation mask is obtained by a majority vote.

**Fig 2:**
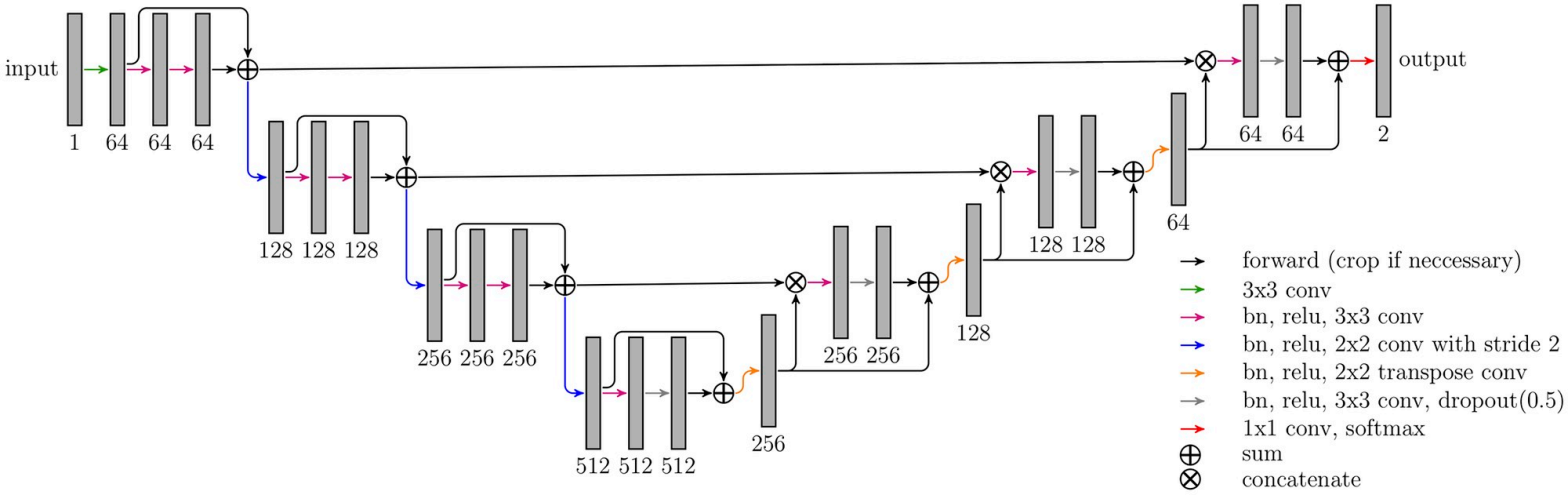
Architecture of CNN models employed in the Liver-Net method. The numbers denote the feature map count.

Our segmentation method Liver-Net employs three orthogonal 2D CNN models trained with axial, coronal and sagittal image patches (see Fig 1) [15]. Each of the CNN models is of a U-net-like [16] architecture working on four resolution levels and has a receptive field of 93 × 93, which was empirically found to provide enough context information for liver segmentation (see Fig 2). The models use convolutional layers with 3 × 3 kernel size. Downscaling (upscaling) is performed via strided (transposed) 2 × 2 convolutions. Each convolution is followed by a batch normalization to speed up the convergence and ReLU nonlinearity. Short skip connections were added to improve the gradient flow throughout the network [17]. Additionally, in the upscaling path, dropout layers were used to avoid overfitting. Hard prediction outputs of the three models are combined via majority vote to produce the final segmentation mask.

#### Training

CNN models were trained using 2D image patches (228 × 228) and a mini-batch size of 16. Input was padded reflectively with 45 pixels on each side to account for convolutions computed in the “valid” mode. Models were trained using the soft dice loss function with the Adam optimizer (10^−4^ learning rate). Model quality was evaluated every 500 iterations on the validation set using the Jaccard coefficient. The training was stopped if the model quality had not improved for 20 validations.

#### Data preprocessing

The images fed into the models were resampled to 2 mm isotropic voxel size and normalized by a linear mapping of the intensities between the 2^nd^ and 98^th^ percentile of each MR image to the [0, 1] range. Random rotations α[deg]∼N(0,10) and random intensity shifts x∼N(0,0.1) were applied to augment the training set.

### Experiments

#### Comparison with manual routine segmentations

To evaluate the segmentation quality of our automatic method, we asked one radiologist Rad (23-year radiology experience) and two radiology residents: Res1 (3-year radiology experience), Res2 (3-month radiology experience) to delineate livers for 21 test patients according to routine clinical standards employed for SIRT planning and to measure the required time. The segmentations were performed using basic contouring software [18]. The automatic and routine segmentations were evaluated against the reference segmentations.

#### Error analysis of the automatic method

To analyze errors of the automatic method, we asked another radiology resident Res3 (2-year radiotherapy, 1-year radiology experience) and one radiologist assistant RA (10-year contouring experience) to correct major errors of the automatically generated liver masks and to measure the correction time. The corrections were done using the same contouring tool as for the routine segmentations. To measure the impact of the corrections, we compared the automatic and corrected liver masks with the references.

Before the study was conducted, all observers participating in this study were trained to use the contouring tool used for manual segmentation and corrections.

#### Inter-observer variability

To assess the influence of the automatic liver segmentation on the inter-observer variability of the estimated liver volume, we compared the inter-observer variability of the manual routine segmentations with the corrected segmentation masks.

### Evaluation metrics

#### Segmentation quality

The segmentation quality was measured using complementary metrics computed in 3D. We used the Dice index (DICE) as an overlap-based metric, the relative volume error (RVE) as a volume-based error measure and the mean surface distance (MSD) and the Hausdorff distance (HD) as surface-based metrics. DICE between two binary objects is defined as:

$$\begin{array}{c} \text{DICE}\left(X,Y\right)=\frac{2|X\cap Y|}{\left|X|+|Y\right|} \end{array}$$ (1)

We define RVE as:

$$\begin{array}{c} \text{RVE}\left(X,Y\right)=\frac{|V_{X}-V_{Y}|}{V_{Y}}\cdot 100\% \end{array}$$ (2)

where *V*_*X*_ and *V*_*Y*_ are the volumes of the test object and reference object, respectively. MSD and HD are defined as follows:

$$\begin{array}{c} \text{MSD}\left(X,Y\right)=\frac{1}{2N}(\sum_{x\in X}\min_{y\in Y}d\left(x,y\right)+\sum_{y\in Y}\min_{x\in X}d\left(x,y\right)) \end{array}$$ (3)

$$\begin{array}{c} \text{HD}\left(X,Y\right)=max\{\underset{x\in X}{\sup}\underset{y\in Y}{\inf}d\left(x,y\right),\underset{y\in Y}{\sup}\underset{x\in X}{\inf}d\left(x,y\right)\} \end{array}$$ (4)

where *d*(*x*, *y*) is the Euclidean distance between points *x* and *y*. To analyze how many slices had to be corrected, we computed a percentage of corrected slices (CS):

$$\begin{array}{c} \text{CS}=\frac{n_{c}}{n_{t}}\cdot 100\% \end{array}$$ (5)

where *n*_*c*_ denotes count of corrected slices and *n*_*t*_ denotes count of slices occupied by the automatic liver mask.

#### Inter-observer variability

The variability among users was measured as the average of absolute inter-observer RVE differences. The consistency analysis of the volume estimation was performed using the intraclass correlation coefficient (ICC) [19]. We used the two-way random, single measure model ICC(2,1).

Differences were tested for statistical significance using the Wilcoxon signed-rank test at 0.01 level.

## Results

The CNNs constituting the Liver-Net were trained in 3h, 1.5h and 6h for the axial, coronal and sagittal model, respectively. Time measurements for training as well as for prediction were done on a desktop PC (Intel Core i7-4770K, 32 GB RAM, NVIDIA Titan Xp).

### Comparison with manual routine segmentations

**Fig 3:**
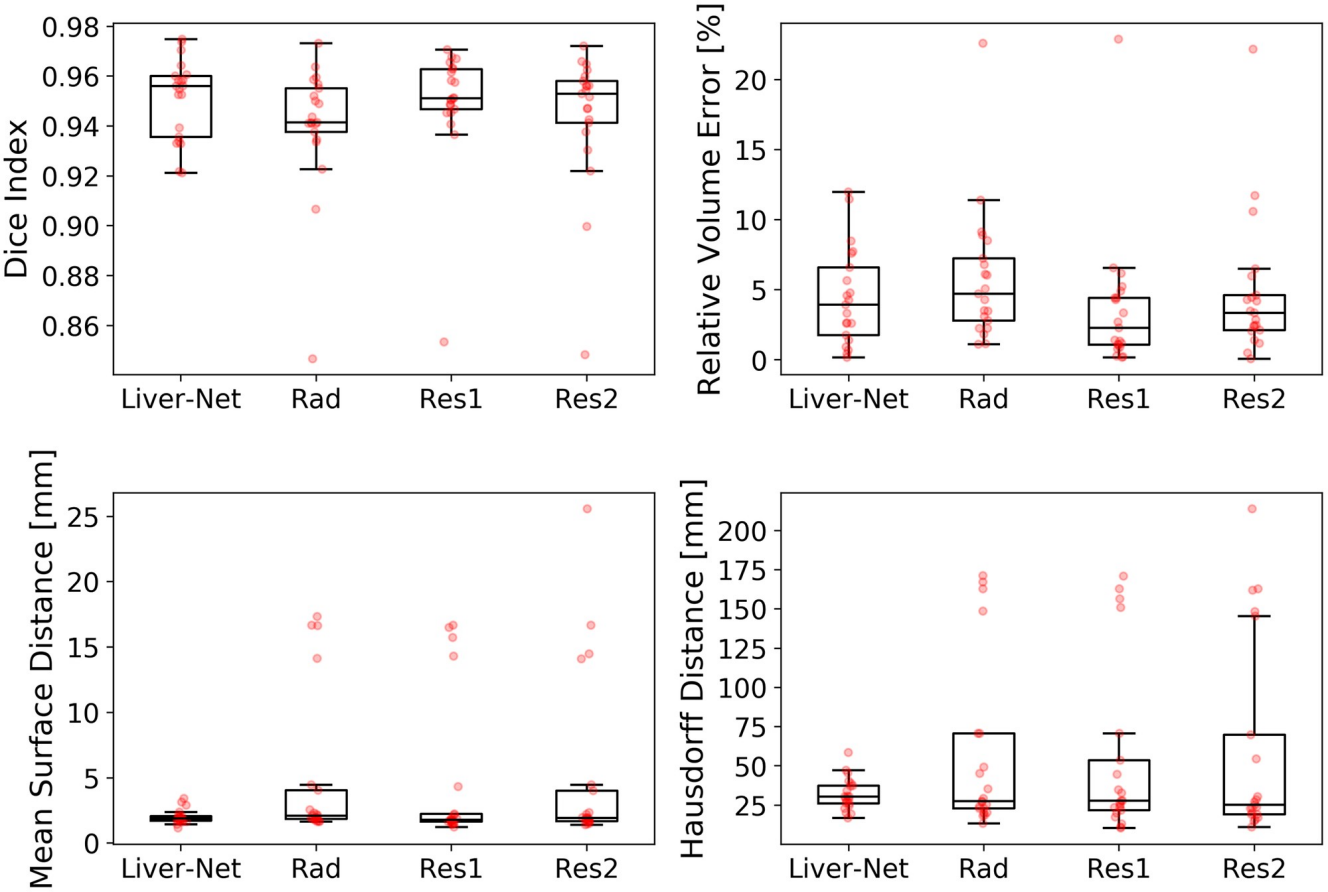
Box plots showing segmentation quality of Liver-Net and that of manual routine segmentations.

**Fig 4:**
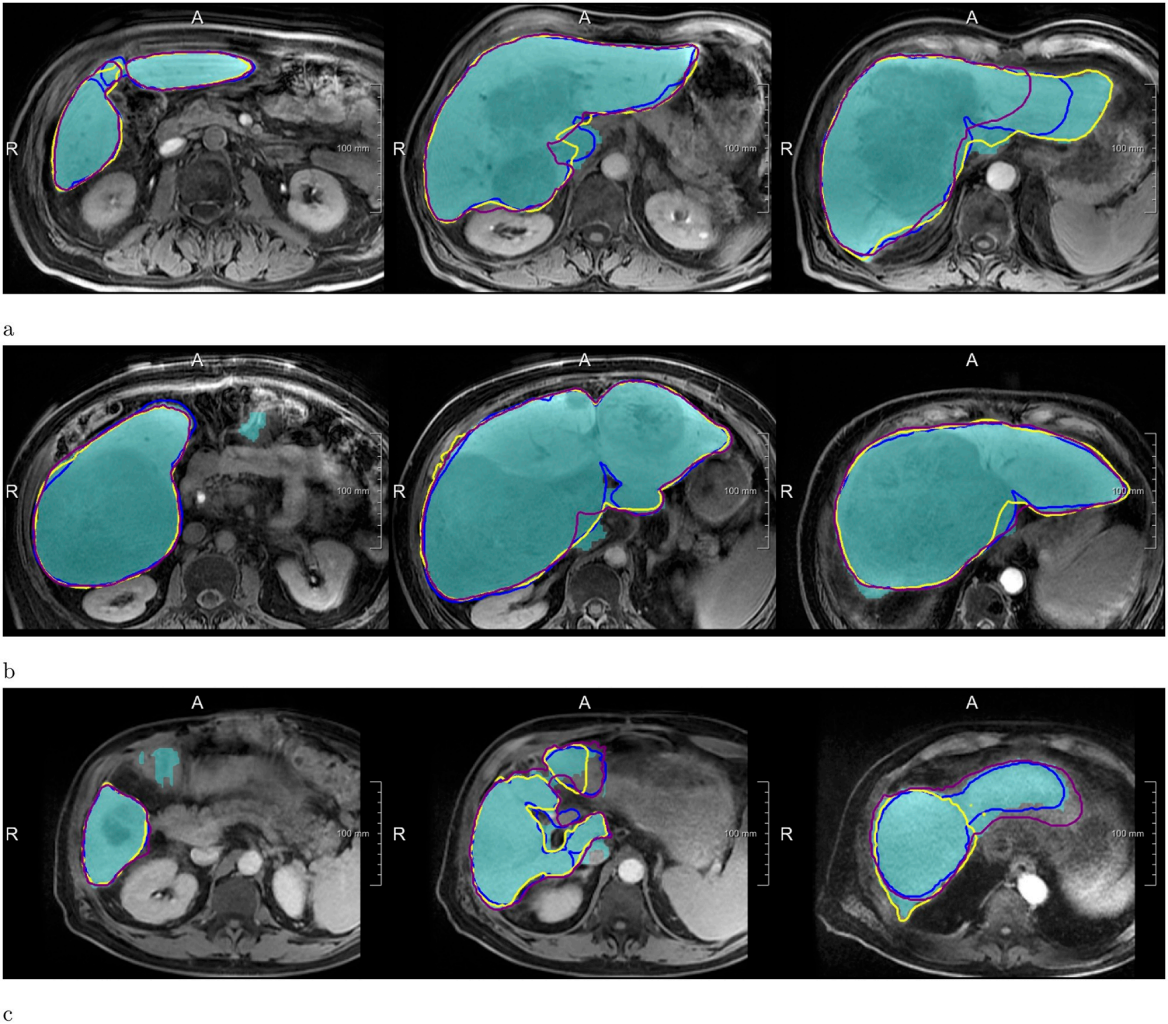
Examples of segmentations produced by the Liver-Net (cyan) compared to three routine delineations by Rad (blue), Res1 (yellow) and Res2 (purple). (a) Case where Liver-Net was more consistent with the reference than the routine segmentations: 0.70% (0.96), 8.89% (0.94), 6.56% (0.95) and 11.7% (0.92) for RVE (DICE) for the automatic method and the routine segmentations by Rad, Res1 and Res2, respectively. (b) Case where automatic and routine segmentations were very similar: 2.62% (0.97), 2.27% (0.97), 0.87% (0.97) and 0.09% (0.97). (c) Case where automatic liver mask had the biggest RVE: 8.49% (0.94), 2.24% (0.94), 0.80% (0.95) and 5.97% (0.94).

The quality of automatic liver masks was on par with manual ones according to the mean DICE: 0.95 for the automatic and 0.94, 0.95, and 0.94 for the manual segmentations (see Table 1 and Fig 3). The mean RVE of the routine segmentations was 5.8%, 3.6%, and 4.7% compared to 4.5% achieved by our method. For the surface distance metrics, our method resulted in the lowest errors (average of 2.0 mm and 32 mm for MSD and HD, respectively). No differences in metric values between the automatic and routine segmentations were significant. Our method needed on average 20 ± 7 s per case, whereas the routine segmentations took 10 ± 4 min. Fig 4 shows representative examples of automatic results compared to routine segmentations.

**Table 1:** Evaluation results (mean ± standard deviation) for the Liver-Net and manual routine segmentations by a radiologist (Rad) and two residents (Res1, Res2) when compared to the reference.

	Liver-Net	Rad	Res1	Res2
DICE	**0.95** ± **0.02**	0.94 ± 0.03	**0.95** ± **0.02**	0.94 ± 0.03
RVE [%]	4.5 ± 3.5	5.8 ± 4.8	**3.6** ± **4.9**	4.7 ± 5.0
MSD [mm]	**2.0** ± **0.5**	4.9 ± 5.7	4.5 ± 5.7	5.1 ± 6.7
HD [mm]	**32** ± **10**	57 ± 55	54 ± 55	60 ± 64

Best results according to the mean are indicated in bold.

### Error analysis of the automatic method

**Fig 5:**
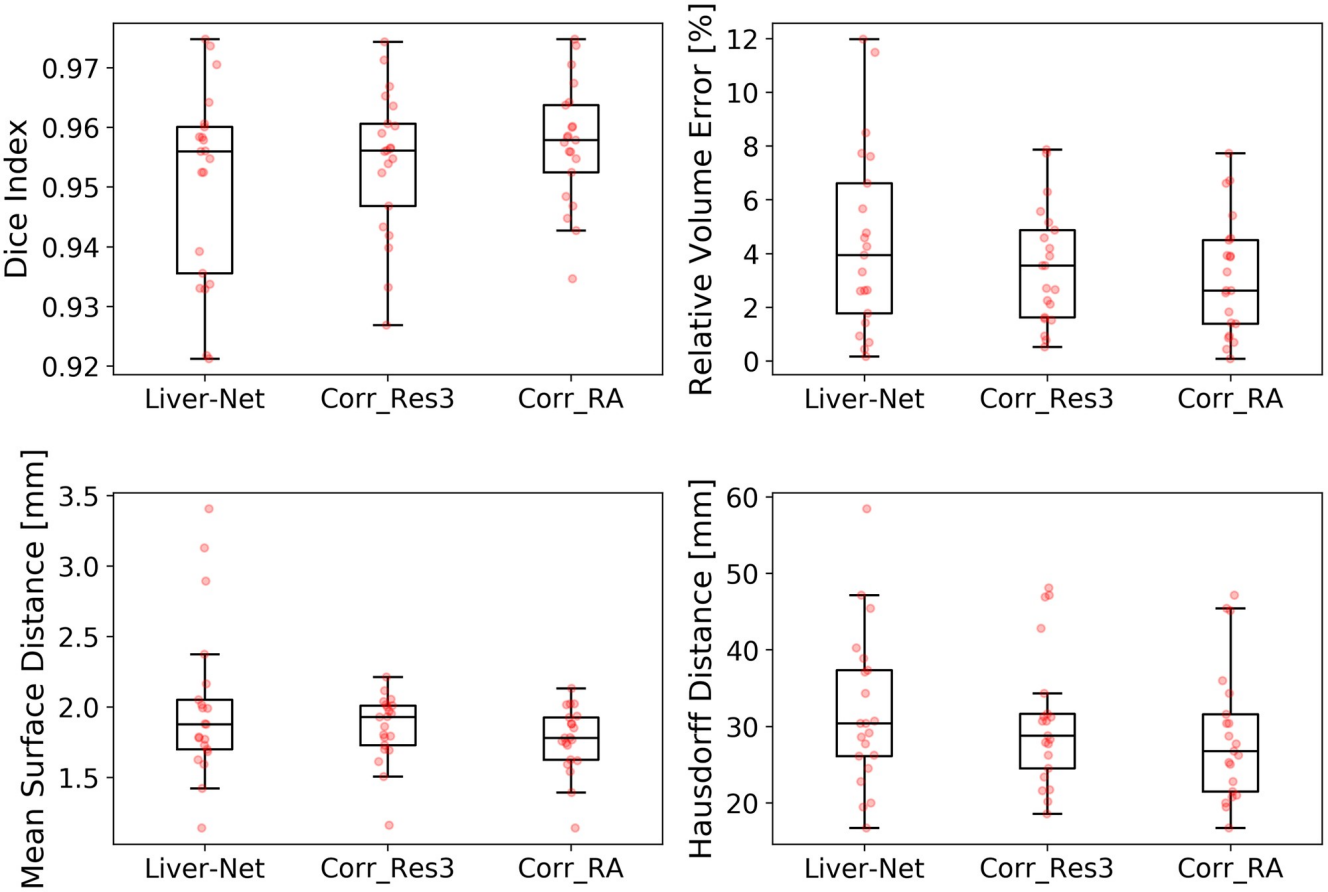
Box plots showing segmentation quality of the Liver-Net masks and their corrections done by Res3 and RA.

**Fig 6:**
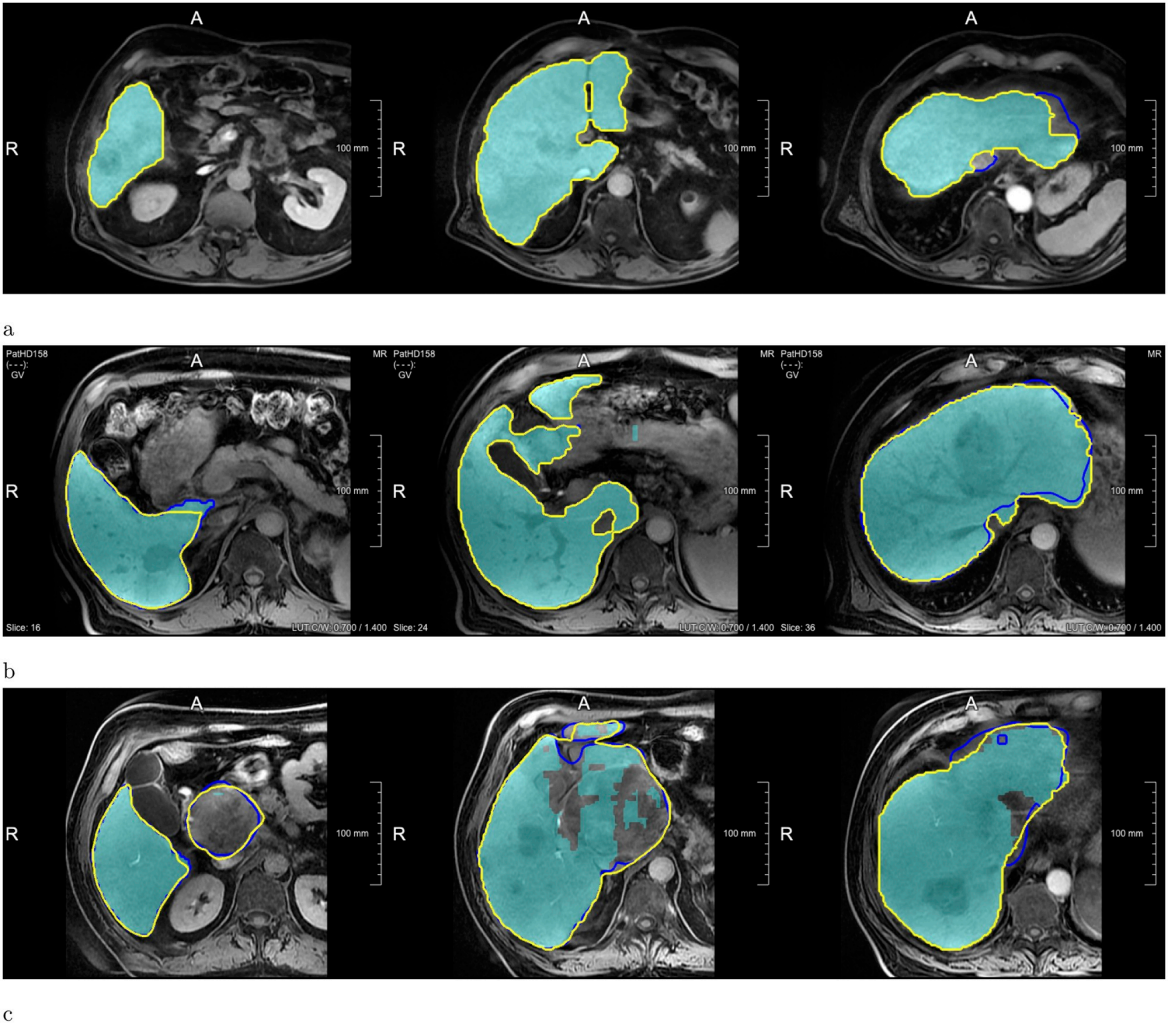
Examples showing how the automatic Liver-Net results (cyan) were corrected. The contours denote corrected liver masks by Res3 (blue) and RA (yellow). (a) Case with minor or no corrections: 1.42% for the Liver-Net liver masks, 1.51% (4.3%) and 1.42% (0%) RVE (CS) for the corrections by Res3 and RA, respectively. (b) Case where less than half of all slices were corrected: 5.66%, 5.57% (47.6%) and 5.40% (35.7%). (c) Case where most of the slices were corrected by all observers: 11.48%, 1.62% (74.0%) and 0.10% (78.3%).

Res3 and RA corrected on average 38% and 25% of slices, respectively, which led to improvements in all error metrics (see Table 2 and Fig 5). Liver masks corrected by the radiologist assistant scored significantly better than the automatic ones according to DICE (p = 0.001), RVE (p = 0.004), and MSD (p = 0.002), when compared with the high-quality reference. The corrections took 2.3 ± 1.7 min for Res3 and less than 2 min for RA. Examples with no or minimal corrections needed as well as ones requiring many adjustments are illustrated in Fig 6.

**Table 2:** Evaluation results (mean ± standard deviation) for the Liver-Net and for the liver masks corrected by Res3 and RA.

	Liver-Net	Corr_Res3	Corr_RA
DICE	0.95 ± 0.02	0.95 ± 0.01	**0.96** ± 0.01*
RVE [%]	4.5 ± 3.5	3.5 ± 2.2	**3.1** ± 2.2*
MSD [mm]	2.0 ± 0.5	1.9 ± 0.2	**1.8** ± 0.2*
HD [mm]	32 ± 10	31 ± 9	**29** ± **9**
CS [%]	n/a	38 ± 30	**25** ± **28**

Best results according to the mean are indicated in bold.

*Significant difference when compared to Liver-Net.

### Inter-observer variability

**Fig 7:**
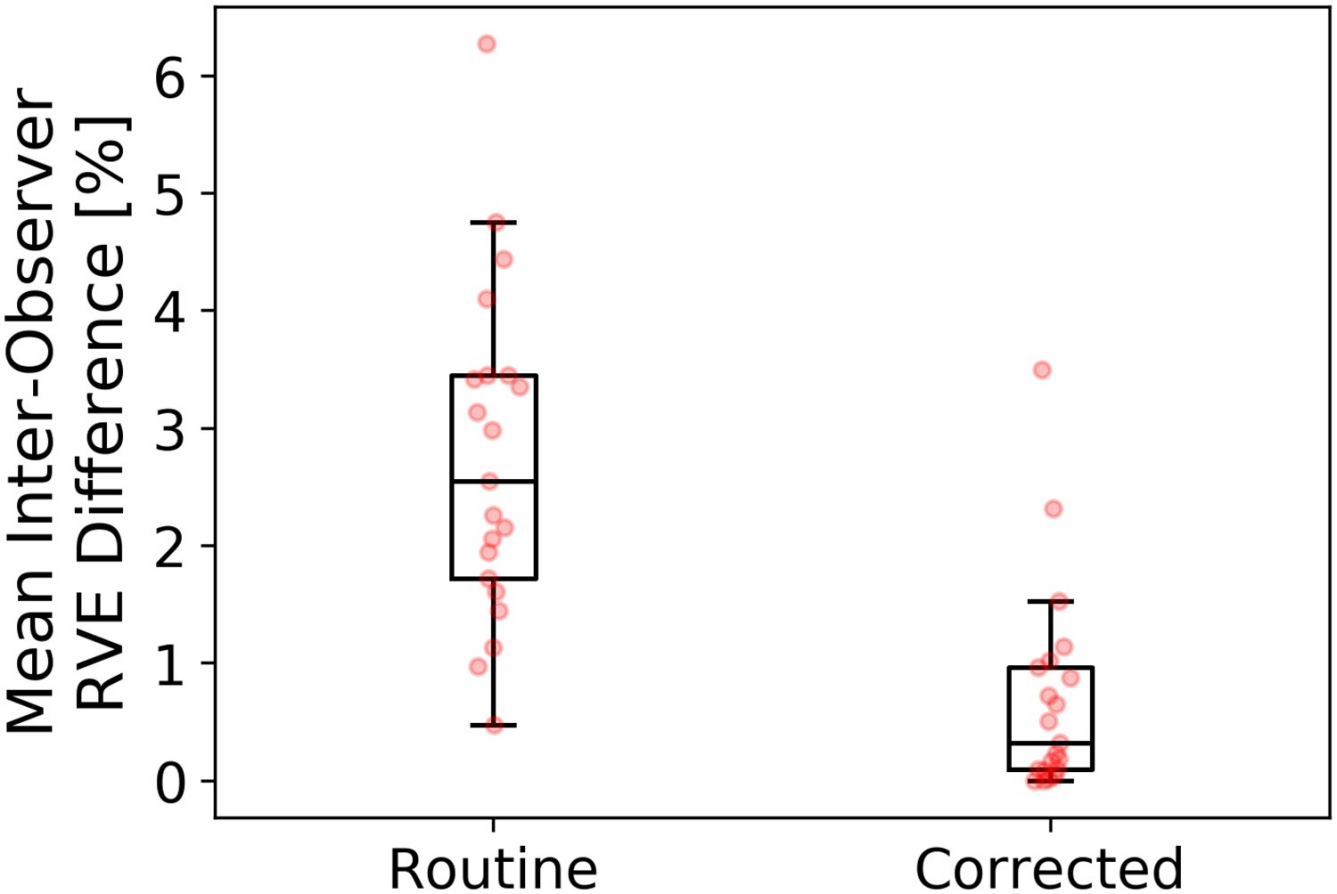
Box plots showing mean absolute inter-observer relative volume difference for routine and corrected automatic segmentations. See Table 3 for RVE values of all observers.

The average per-case absolute inter-observer RVE difference was 2.75 ± 1.41% for the manual routine segmentations, which was significantly higher (p = 0.0002) compared to 0.69 ± 0.88% for corrected automatic liver masks (Fig 7). Table 3 lists RVE values for each patient and each observer. The ICC(2,1) with a 95% confidence interval was 0.989 (0.978, 0.995) and 0.999 (0.998, 1) for manual and corrected segmentations, respectively.

**Table 3:** Per patient relative volume error for routine and corrected automatic segmentations.

	Routine			Corrected	
	Rad	Res1	Res2	Res3	RA
Patient1^†^	8.89%	6.56%	11.73%	4.20%	0.70%
Patient2	8.52%	4.44%	1.39%	4.59%	3.87%
Patient3	1.84%	4.42%	2.45%	5.57%	5.41%
Patient4	1.15%	6.19%	2.07%	2.25%	4.56%
Patient5	4.72%	0.24%	3.47%	0.93%	0.93%
Patient6	2.81%	1.09%	4.47%	6.30%	6.62%
Patient7	3.49%	0.26%	1.17%	3.56%	3.32%
Patient8	3.51%	1.10%	3.37%	4.88%	3.91%
Patient9	2.27%	0.87%	0.09%	2.11%	2.62%
Patient10	22.62%	22.89%	22.18%	1.62%	0.09%
Patient11	7.24%	2.69%	2.12%	0.78%	0.87%
Patient12	3.10%	0.17%	2.40%	7.73%	7.73%
Patient13	2.24%	0.79%	5.97%	7.86%	6.72%
Patient14	6.06%	4.93%	4.61%	3.56%	2.54%
Patient15	11.39%	5.24%	6.50%	0.52%	0.43%
Patient16	5.07%	3.37%	4.29%	5.15%	4.51%
Patient17	1.13%	1.34%	4.21%	2.66%	2.62%
Patient18*	6.79%	1.17%	10.58%	2.71%	1.83%
Patient19	9.14%	4.28%	2.49%	1.57%	1.38%
Patient20	6.12%	1.42%	2.87%	1.51%	1.42%
Patient21	4.31%	2.30%	0.49%	3.91%	3.93%

Case with the highest inter-observer variability among manual routine* and corrected^†^ segmentations: 6.27% and 3.50%, respectively.

## Discussion

Our Liver-Net method delivered segmentations which were on par with the manual routine segmentations. In case an observer corrected the automatic liver segmentation, the contours and subsequent volume measurement become less subjective than manual segmentations from scratch. The automatic suggestions may help to achieve segmentations more consistent with guidelines used to segment the training data. For example, in the case of Patient10 from Table 3, routinely segmented livers did not include a large lesion in the liver mask, which was not the case for the reference and both corrected segmentations (see Fig 6). Only RA, who also created reference segmentations, was able to achieve a significant quality improvement of corrected masks according to DICE, RVE, and MSD when compared to the automatic Liver-Net segmentations. The consistency among observers measured as ICC(2,1) for manual (0.989) and corrected (0.999) segmentations was excellent and similar to the inter-observer correlation reported for CT (0.997) [20].

**Fig 8:**
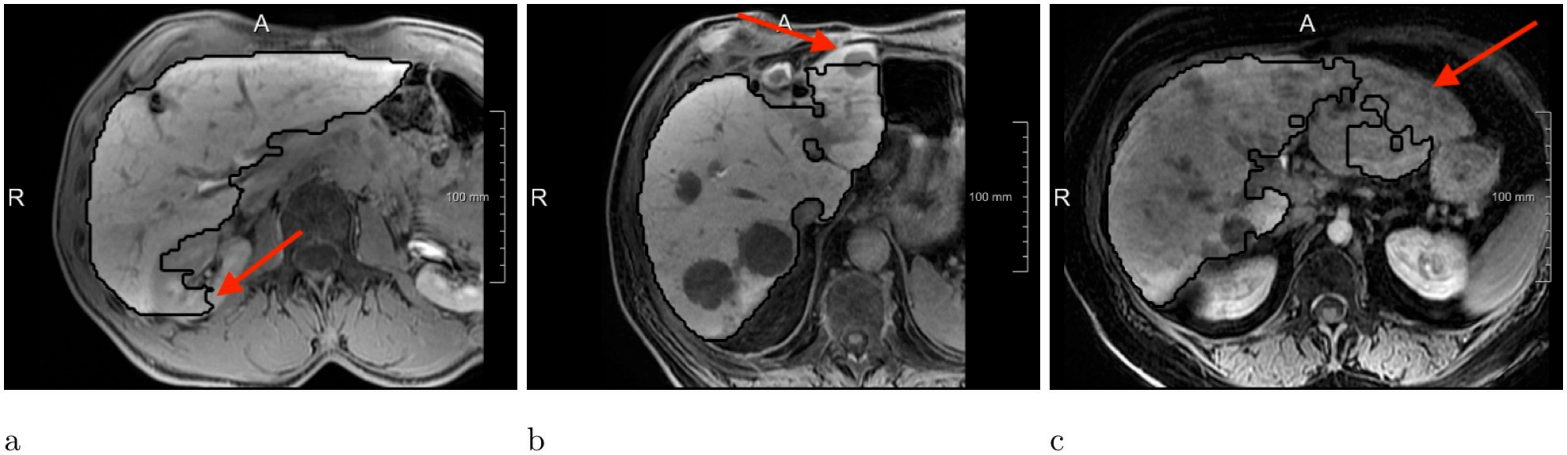
Most common error types of the Liver-Net approach. (a) Leakage into surrounding structure of similar image intensity. (b) FNs due to lesions located near the organ boundary. (c) Liver underestimation caused by a big inhomogeneous lesion.

We asked an expert radiologist to analyze visually how the liver masks produced by Liver-Net were corrected by observers and the following factors causing major segmentations were identified (see Fig 8 for error examples):

- Contrast changes causing FPs and FNs due to lesions located near the organ surface or contrast enhanced vessels.
- Large inhomogeneous lesions leading to liver underestimation.
- Similar intensities of surrounding nearby organs cause FPs (e.g., kidneys and stomach).

### Comparison with other methods

The segmentation quality of the Liver-Net evaluated using a challenging data set consisting of pathological livers with numerous tumors was 0.95 according to DICE, which is better than other methods in the literature reporting DICE in the 0.87-0.91 range [4–7]. Unfortunately, a direct comparison is not possible, as different datasets were used for method optimization and evaluation.

**Fig 9:**
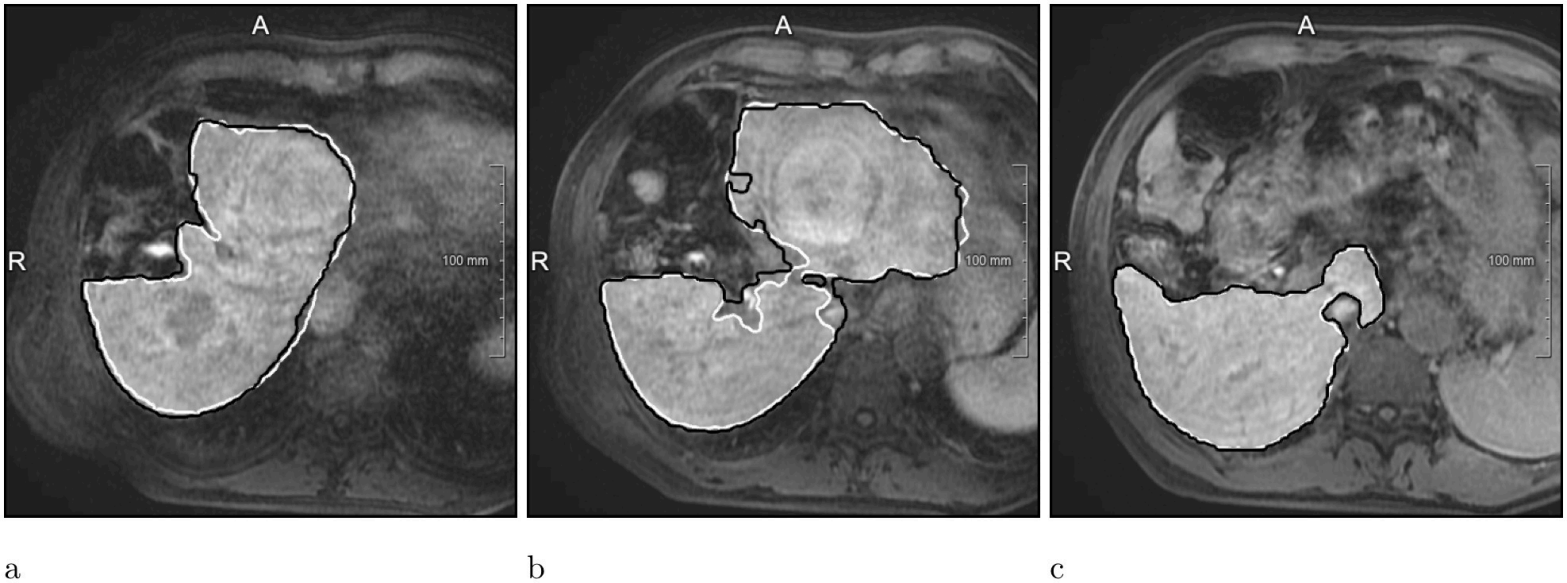
Resected liver case, where the Liver-Net method (black) produced segmentation consistent with the reference (white): 3% RVE and 0.95 DICE.

Liver-Net, in contrast to approaches employing shape models [4–6], does not contain an explicit knowledge about plausible liver forms. Therefore, in some cases, it produces segmentations that do not completely resemble a liver (Fig 8a). On the other hand, the lack of a shape prior allows for a correct segmentation of livers strongly deviating in form from a normal liver shape (e.g., resected liver, Fig 9). Compared to the approach of Christ et al. [7] where only one axial 2D CNN was used, Liver-Net uses three orthogonal models, which allow our method to exploit the 3D context information of volumetric MR images.

### Study limitations

The test set size (21 images) should be taken into account when considering the conclusions of this work, as the small sample size limits the accuracy of the statistical analysis.

## Conclusion

We presented a validation of a fully automatic method dubbed Liver-Net for liver segmentation in MRI based on three orthogonal neural networks and its corrected masks. The comparison with manual routine segmentations provided by three clinical users showed that Liver-Net produces liver masks of a comparable quality according to Dice index, relative volume error, mean surface distance, and Hausdorff distance. Error analysis of the Liver-Net showed that, on average, 32% of slices had to be corrected, which required on average 2 minutes of interaction time. Corrected liver masks led to a significantly lower inter-observer variability when compared to manual segmentations from scratch (0.69% vs. 2.75% for mean per-case absolute inter-observer RVE difference and 0.999 vs. 0.989 for the intra-class correlation coefficient). We identified the following factors as sources of major errors of our method: contrast changes close to organ boundary, inhomogeneous lesions and similar appearance of surrounding structures. Successful automated elimination of these errors would most probably lead to a substantial improvement in segmentation quality. One possible solution to this may be the extension of the training dataset with cases causing the above-mentioned errors. Additionally, a hard example mining strategy could be employed, which concentrates the learning process on the most difficult cases.

Future research directions include usage of 3D neural networks exploiting the full volumetric context information, GAN models for generation of training data [21] and combination of CNNs with statistical shape knowledge [22].
